# Grima: A Distinct Emotion Concept?

**DOI:** 10.3389/fpsyg.2017.00131

**Published:** 2017-02-03

**Authors:** Inge Schweiger Gallo, José-Miguel Fernández-Dols, Peter M. Gollwitzer, Andreas Keil

**Affiliations:** ^1^Facultad de Ciencias Políticas y Sociología, Departamento de Psicología Social, Universidad Complutense de MadridMadrid, Spain; ^2^Facultad de Psicología, Departamento de Psicología Social y Metodología, Universidad Autónoma de MadridMadrid, Spain; ^3^Motivation Lab, Psychology Department, New York UniversityNew York, NY, USA; ^4^Social Psychology and Motivation, Department of Psychology, University of KonstanzKonstanz, Germany; ^5^Center for the Study of Emotion and Attention, University of FloridaGainesville, FL, USA

**Keywords:** disgust, grima, aversion, asco, implementation intentions

## Abstract

People experience an unpleasant sensation when hearing a scratch on a board or plate. The present research focuses on this aversive experience known in Spanish as ‘grima’ with no equivalent term in English and German. We hypothesized that this aversive experience constitutes a distinctive, separate emotional concept. In Study 1, we found that the affective meaning of ‘grima’ was closer to *disgust* than to other emotion concepts. Thus, in Study 2 we explored the features of *grima* and compared them with *disgust*. As *grima* was reported to be predominantly elicited by certain auditory stimuli and associated with a distinctive physiological pattern, Study 3 used direct measures of physiological arousal to test the assumption of a distinctive pattern of physiological responses elicited by auditory stimuli of grima and disgust, and found different effects on heart rate but not on skin conductance. In Study 4, we hypothesized that only participants with an implementation intention geared toward down-regulating grima would be able to successfully weaken the grima- but not disgust- experience. Importantly, this effect was specific as it held true for the grima-eliciting sounds only, but did not affect disgust-related sounds. Finally, Study 5 found that English and German speakers lack a single accessible linguistic label for the pattern of aversive reactions termed by Spanish speaking individuals as ‘grima’, whereas the elicitors of other emotions were accessible and accurately identified by German, English, as well as Spanish speakers.

*HOTSPUR: Marry*,

And I am glad of it with all my heart:

I had rather be a kitten and cry mew

Than one of these same metre ballad-mongers;

*I had rather hear a brazen canstick turn’d*,

Or a dry wheel grate on the axle-tree;

*And that would set my teeth nothing on edge*,

Nothing so much as mincing poetry:

’Tis like the forced gait of a shuﬄing nag.

**Shakespeare (1596), *Henry IV, Part I***

## Introduction

Shakespeare’s quotation of Henry IV graphically illustrates that some auditory stimuli possess physical characteristics that directly trigger aversive responses. Such is the case of screeching or creaking (i.e., those high-pitched, shrill sounds like the noise of a piece of chalk on a blackboard, a fork scratching a plate, or the scraping of fingernails on a surface). These auditory stimuli produce an aversive emotional reaction in ways that have not yet been extensively characterized.

Whereas some Western languages, English for example, seem to lack a specific term for this experience to high-pitched and shrill sounds, Spanish speakers refer to it as ‘grima’^1^. This peculiarity has allowed us to assess (a) the conceptual, behavioral, and physiological features of this aversive experience, and (b) its differences with respect to a term that also alludes to emotional aversive reactions (i.e., ‘asco’ or ‘disgust’). As ‘asco’ was found to have the closest affective meaning to ‘grima’ in a first study, we aimed to show across four studies that the Spanish ‘grima’ is backed by a well-delimited everyday concept which alludes to distinct physiological correlates, and requires specific regulatory strategies different from those involved in the visual (e.g., Marzillier and Davey, 2005; Adolph and Pause, 2012; Sherman et al., 2012), tactile, olfactory, or gustatory (e.g., Croy et al., 2013) aversive experiences labeled as ‘disgust’ (‘asco’ in Spanish)^2^.

Many contemporary emotion approaches consider emotions as being each qualitatively different from another. From this point of view, universal basic emotions are associated with specific physiological and behavioral correlates. Thus, each emotion is a distinct member of the family of basic emotions. In contrast, appraisal theories and psychological construction models question or challenge the view of emotions as a limited repertoire of discrete categories. According to appraisal theories it is the evaluation (“appraisal”) of the environment which elicits and serves to differentiate between emotions (e.g., Scherer, 1999; Ellsworth and Scherer, 2003). Thus, different appraisals are assumed to elicit different emotions (Roseman and Smith, 2001). Though appraisal theorists differ in their appreciation of the number of appraisal patterns and thus of different emotional states (Scherer, 2000), most accept a vast number of emotional states (Ellsworth, 2013). The underlying assumption is that one single situation can cause different emotions, as each situation can be interpreted (i.e., appraised) in multiple ways depending on the individual (Roseman and Smith, 2001). Similarly, different circumstances can cause feeling one and the same emotion when they are elicited by the same underlying appraisal pattern.

Psychological construction models (e.g., Russell, 2003) endorse the concept of appraisal but challenge the view of emotions as natural kinds. Psychological constructionism does not conceive of emotions as unitary, objective phenomena with a clear definition in terms of necessary and sufficient characteristics (i.e., latent construct called ‘disgust’, ‘anger’, and so on), but as a set of independent experiential, physiological, and behavioral reactions (“emotional episode”; Russell, 2003, 2014) that can occur simultaneously or in close temporal proximity in some specific circumstances and may have causal relationships among themselves. Thus, psychological constructionism approaches emotions as entailing two related but independent phenomena: on the one hand, the co-occurrence of the different objective components of an emotional episode, and, on the other hand, the cognitive categorization of this event. A key point in which basic emotion approaches and psychological constructionism differ is the assumption of relativity of the categories of emotions and the existence of important differences in the way in which people from different cultures classify emotional episodes into certain emotion terms

Growing research supports consistently the assumption that *disgust* may not be one unitary, homogeneous concept. Nabi (2002), for example, has suggested that the lay meaning of ‘disgust’ encompasses not only *disgust*, but *anger* too, and thus differs from the theoretical meaning of ‘disgust’. That the word ‘disgust’ can also refer to one type of disgust but not others has been supported by Yoder et al. (2016) with respect to facial expressions. They found that different types of disgust elicitors such as death, animals, or hygiene were systematically associated with different types of facial expressions (standard disgust, sickness, and sadness in Study 1, plus anger in Study 2). More specifically, the sick face was more often associated with physical disgust than was the standard disgust face, while the standard disgust face (or an anger face; Study 2) was primarily associated with moral disgust.

The lack of homogeneity also has clear implications when it comes to translating emotion concepts (overview by Russell, 1991; see also Mesquita and Frijda, 2011). A recent study by Han et al. (2015) showed that English speakers use the word ‘disgust’ to refer to distaste, pathogen-containing substances, blood and injury, inappropriate sexual events, and moral violations. In contrast, Korean speakers use the translation of ‘disgust’ only to refer to some of them (i.e., distaste, pathogen-containing substances, as well as blood and injury), while the translation equivalent in Malayalam does not refer to any of them. Thus, in line with Wierzbicka (1999), who stated that there might not be translation equivalents of the word ‘disgust’ in other languages, Han et al.’s (2015) results suggest that this happens with the translation of ‘disgust’ into Malayalam. The same is true for the Chinese equivalent of the term ‘disgust’: a lack of adequate translation was found by Barger et al. (2010), and has been recently supported by data from (Schweiger Gallo et al., unpublished manuscript), who found different internal structures of the concept of *disgust* in Spain, Germany, the USA, China, and Palestine. In sum, this lack of equivalence across languages is consistent with the argument that the English word ‘disgust’ can refer to different emotional reactions.

As compared to those cases in which there are no equivalent single words or concepts for some experiences, for example, as in English for the German ‘Schadenfreude’ or the Japanese ‘itoshii’ (Russell, 1991), people in some cultures may rely on different colloquial expressions to refer to a certain emotional experience. In the present research, we suggest that this is the case with the Spanish grima. In fact, though people in different cultures share this experience when they hear, for example, a scratch on a board or a plate, we suspect that not all of them refer to it by using a single term. Support for our hypothesis is provided by the only literature based on this experience, where it is described as having teeth set on edge (Delin and Winefield, 1973, 1977), as well as by searching the Internet, where people refer to it in English using expressions such as ‘it makes me grue’, ‘ibby gibbies’, and so on; and in German as ‘einem den Rücken eiskalt runterlaufen’ (’it gives someone the creeps’) and ‘Gänsehaut bekommen’ (goosebumps).

As Spanish speakers make common use of the word ‘grima’, the reasons for analyzing this experience are manifold: first of all, from a conceptual point of view, mapping its content is expected to shed light onto its elicitors, as well as the feelings, expressions, physiological and behavioral responses associated with the experience. In addition, assessing the content of *grima* also allows us to analyze its relationship with respect to other emotional experiences. In fact, by specifically comparing *grima* and *disgust* we can provide the first empirical description of *grima* as compared to *disgust*. This is of importance, as past studies assessing emotions, and specifically disgust, might have been tapping features pertaining to the experience of grima. Finally, the existence of a specific term for this reaction raises a number of interesting theoretical questions: is grima an emotional experience on its own or a mere reflex reaction? One possible answer to this question might be based on whether or not grima involves cognitive processes. In fact, introducing a cognitive process such as the down-regulation of an emotional experience would allow for testing whether grima might better be regarded a mere reflex reaction rather than an emotional experience *per se*. Further questions include: does grima, as a form of aversive reaction based on distinct sensorial input, also have some distinct physiological or behavioral correlates? And does the existence of the term lead Spanish speakers to perceive this aversive experience differently, as compared to those speakers, for example English speakers, who seem to lack an equivalent term?

We take advantage of the Spanish term ‘grima’ to explore the constitutive features of the concept behind this term; a task that would be hard to accomplish by focusing on those languages in which participants do not have a specific term for this aversive reaction. In this regard, we hypothesize that grima is a distinct emotional episode with most of its components being probably universally shared. Thus, even though grima is characterized in Spanish via a dedicated term, it is probably also experienced in other cultures without a cognitive representation for this experience. In order to differentiate grima from other emotional experiences, we first explore which concept of emotion has the closest affective meaning with regard to *grima* (Study 1) and the content universe of *grima* (Study 2). Furthermore, we analyze whether the conceptual differentiation between *grima* and *disgust* observed in Studies 1 and 2 is supported by a parallel differentiation of physiological responses associated with the experience of grima and disgust (Study 3). Since we assume that grima constitutes a distinct, universally shared emotional experience, we expect different physiological profiles in both heart rate (HR) and skin conductance response (SCR). In Study 4, we assess whether the differentiation between *grima* and *disgust* also holds true when it comes to the differential regulation of one of them (i.e., down-regulating grima, but not disgust). Since a self-regulation strategy known as implementation intentions (i.e., if-then plans) has been found in previous research to be effective in down-regulating unpleasant emotions such as disgust or fear (e.g., Schweiger Gallo et al., 2009), participants in Study 4 are either assigned to a goal intention condition or an implementation intention condition and asked not to feel grima. Based on previous research on implementation intentions, only implementation intention participants are expected to be able to down-regulate their grima-experience, as compared to goal intention participants. Further, we expect implementation intention participants to selectively down-regulate their experience to grima- but not disgust-sounds. Finally, Study 5 tests whether the affective aversive reaction evoked by high-pitched and squeaking noises is conceptually assimilated to other aversive reactions in those languages lacking a single accessible linguistic label to describe the aversive experience of what is called ‘grima’ in Spanish. Based on the observation that German and English speakers recur to a variety of metaphorical descriptions to refer to this experience, we expect only Spanish speakers to label the experience with a unique term.

## Study 1: Delineating ‘Grima’ from Other Emotion Terms

In this Study, we aimed at delineating ‘grima’ from other emotion terms in order to test which concept of emotion has the closest affective similarity. Here, the so-called Affect Grid was deemed as the ideal method to test the location of different emotional experiences in an affective space described by the dimensions of pleasure-displeasure and arousal-sleepiness (see Russell et al., 1989).

### Materials and Methods

#### Participants

One hundred and sixteen Spanish undergraduates attending a Spanish university contributed data during class. The average age was 21.7 (*SD* = 2.44). Participants tacitly gave their consent by completing and returning the questionnaire.

#### Materials and Procedure

We administered the Spanish adaptation of the Affect Grid (Russell et al., 1989) developed by Ruiz-Belda (1995). In order to familiarize participants with the Affect Grid, they were asked to read carefully a set of instructions that explained in detail the meaning of the different areas and how to use the grid. Next, participants were asked to rate *disgust* (‘asco’), *fear* (‘miedo’), *sadness* (‘tristeza’), *happiness* (‘alegria’), *grima* and *anger* (‘ira’) along the two orthogonal axes of the Affect Grid, with the x-axis representing pleasure with values between 4 (maximum pleasure) and —4 (maximum displeasure) and the y-axis representing arousal with values between 4 (highest arousal) and –4 (lowest arousal).

### Results

First of all, we calculated the Euclidean distance on the affective space between *grima* and the other concepts of emotion, based on the average ratings on the pleasure and arousal dimensions. Results revealed that closest to *grima* was *disgust* (2.8), followed by *fear* (3.1), *anger* (3.3), *sadness* (3.6), and *happiness* (6.8). Thus, in a next step we computed within-samples *t*-tests, comparing *grima* with *fear*, *anger*, *sadness*, *happiness and disgust*. Results showed that *grima* and *disgust* differed significantly on both the pleasure-displeasure (*M* = –2.37, *SD* = 1.27 and *M* = –3.16, *SD* = 0.86; 95% CI [–1.03, –0.55]), *t*(114) = 6.59, *p <* 0.01, *d* = 0.61, and the arousal-sleepiness dimensions (*M* = 0.50, *SD* = 2.15 and *M* = 1.30, *SD* = 2.47; 95% CI [0.26, 1.32]), *t*(114) = 2.96, *p <* 0.01, *d* = 0.28. The comparisons between *grima* and *anger* (*M* = –2.35, *SD* = 1.28 and *M* = –2.70, *SD* = 1.61; 95% CI [0.00, 0.71]), *t*(114) = 2.01, *p <* 0.05, *d* = 0.19, *sadness* (*M* = –2.35, *SD* = 1.29 and *M* = –2.88, *SD* = 1.25; 95% CI [0.21, 0.84]), *t*(113) = 3.31, *p <* 0.01, *d* = 0.31, and *happiness* (*M* = –2.35, *SD* = 1.28 and *M* = 3.35, *SD* = 1.09; 95% CI [-6.02, -5.37]), *t*(114) = 35.04, *p <* 0.01, *d* = 3.50 were also significant on the pleasure-displeasure dimensions, with the exception of *fear* (*M* = -2.35, *SD* = 1.28 and *M* = –2.63, *SD* = 1.48; 95% CI [–0.05, 0.61]), *t*(114) = 1.68, *p* = 0.1, *d* = 0.16, where the difference was only marginally significant. With respect to the arousal-sleepiness dimension, the comparisons between *grima* and *anger* (*M* = 0.54, *SD* = 2.17 and *M* = 2.90, *SD* = 1.85; 95% CI [–2.82, –1.91]), *t*(114) = 10.26, *p <* 0.01, *d* = 0.96, *sadness* (*M* = 0.55, *SD* = 2.17 and *M* = –1.29, *SD* = 2.55; 95% CI [1.24, 2.45]), *t*(113) = 6.05, *p <* 0.01, *d* = 0.57, *happiness* (*M* = 0.54, *SD* = 2.17 and *M* = 3.17, *SD* = 1.32; 95% CI [–3.12, –2.15]), *t*(114) = 10.77, *p <* 0.01, *d* = 1.07 and *fear* (*M* = 0.54, *SD* = 2.17 and *M* = 2.36, *SD* = 2.13; 95% CI [–2.30, –1.34]), *t*(114) = 7.48, *p <* 0.01, *d* = 0.70 also reached significance.

### Discussion

Results showed that *grima* was evaluated as being less pleasant but more arousing than *disgust*. Given that ‘grima’ and ‘disgust’ had the closest affective meaning, we decided to compare *grima* and *disgust* in the following studies. This comparison was expected to shed more light on the contents and structure of *grima*, as well as the differences and similarities between *grima* and *disgust*.

## Study 2: Content Universe of *Grima*

Taking advantage of the fact that Spanish speaking individuals use the specific term ‘grima’ to refer to the respective aversive reaction, we explored the everyday concept of *grima* in order to collect its most prototypical features. Based on previous prototype research (e.g., Russell and Fehr, 1994; Gregg et al., 2008), we asked participants to generate features of the Spanish concept of *grima.*

### Materials and Methods

#### Participants

One hundred and fifty-five participants filled out a questionnaire either in the classroom or individually. It was assured that for all of the participants, Spanish was their native language, with the exception of one participant whose native language was German, although the participant lived in Spain for almost 33 years. As three participants did not report any situation in which they had experienced grima and one declared not having had the experience, these four participants were excluded. The mean age was 30 years (*SD* = 10.49).

#### Materials and Procedure

Participants were told that we were interested in *grima*. Therefore, participants were first asked to define *grima* and then to list as many situations or objects of *grima* as readily came to their minds. They were asked to stop after about a minute or 10 items. In addition, participants then reported on their typical physical reactions when experiencing grima. At the end, all participants were asked to answer demographic questions.

### Results

Participants’ answers were coded by two independent raters following the procedure by Fehr (1988). First of all, all linguistic units (i.e., single units) were extracted from the responses of the participants. These linguistic units represent separate features (e.g., “negative stimulus for the nervous system, so much that the stomach shrinks. It is related to the hearing and touch” was divided into “negative stimulus for the nervous system,” “stomach shrinks,” “related to the hearing,” and “related to the touch.”) Different linguistic units were grouped if they were grammatical variations of the same word, if they were modified by adjectives, or very similar in their meaning. For example, “noises and squeaking” was composed by linguistic units such as “squeaking with a knife on a plate,” “knife squeaking on a plate,” “fork scratching plates squeaking,” “high-pitched sound such as when the chalk squeaks on a blackboard,” “sound of a chalk on a blackboard,” etc.

Only a negligible number of disagreements occurred as the task required minimal interpretation from the coders. When the coders did not agree, consensus was reached by discussion. We only considered those features that were generated by at least 10% of the participants.

The preliminary extraction yielded 418 linguistic units for the definition of *grima*, which were grouped into 203 features. Of these, 149 were idiosyncratic (i.e., mentioned by only 1 participant). The feature most frequently mentioned was “unpleasant sensation,” (35 participants) followed by “shivering” (20 participants). The same number of participants (18) pointed to “noises/sounds” and “sensation of repulsion.” The final feature mentioned by 17 respondents was “sensation of disgust.”

The preliminary extraction of linguistic units of typical elicitors of *grima* yielded 497 linguistic units, which were grouped into 101 groups of features. Of these, elicitors belonging to noises and squeaking, such as the noise of chalk on a blackboard, were mentioned by 80 participants, followed by scratching or touching with fingernails, mentioned by 52 participants, and scratching or touching of surfaces (mentioned by 40 participants). Importantly, 53%, 34% and 26% of the participants mentioned these features, respectively, which points to their typicality. The animals such as cockroaches or snakes were also mentioned as typical elicitors of *grima* (40 participants), followed by accidents and physical damage (33 participants), sense of touch of objects, such as cotton (32 times), and chewing and biting objects such as wool (22 participants). Elicitors from the moral domain such as racism reached 21 mentions, followed by issues related to fingernails such as biting fingernails (19 participants), fabrics such as cutting cotton, smells such as the smell of vomit (both mentioned by 18 participants), and food, such as rotten food (mentioned by 16 participants).

The typical physical reactions of *grima* included 177 linguistic units, which were sorted into 82 groups of features. Of these, only shivering and goose bumps received a significant number of mentions (72 and 51, respectively).

As participants mentioned disgust (‘asco’) when describing *grima*, we also compared the typical features of *grima* with those of *disgust* by asking eighty Spanish speaking individuals between 16 and 56 years (mean age 25 years; *SD* = 8.54; 38% males) to list as many situations or objects of *disgust* as came readily to their mind as well as to report on their typical physical reactions when experiencing disgust. With respect to the features of *disgust*, 344 linguistic units were extracted and grouped into 64 groups of features. Results showed that the features related to *grima* (noises and squeaking, as well as scratching or touching with fingernails and scratching or touching of surfaces) were not mentioned. The same was true for chewing and biting objects, fabrics and issues related to fingernails. In contrast, the moral domain, smells, and animals were mentioned by 78, 61, and 60 participants, while food, accidents/physical damage, and the specific sense of touch of objects were mentioned by 47, 15, and 2 participants, respectively. Among the 106 reported typical physical reactions it was shivering and goose bumps that were mentioned 9 and 6 times, respectively. Finally, we compared the respective proportions of typical features for *grima* and *disgust* and computed their respective confidence intervals (see **Table 1**). Results revealed significant differences for noises and squeaking, scratching or touching with fingernails, scratching or touching of surfaces, animals, sense of touch of objects, chewing and biting objects, moral domain, fingernails, fabrics, food, and smells, with the exception of accidents/physical damage. With regards to the physical reactions, shivering and goose bumps also differed significantly.

**Table 1:** Comparisons of the respective proportions for *grima* features and *disgust* features (Study 2).

Category	*z*	*p*	LLCI	ULCI	Cohen’s *h*
Noises and squeaking	12.65	0.00	0.28	0.40	1.60
Scratching or touching with fingernails	8.70	0.00	0.17	0.27	1.24
Scratching or touching of surfaces	7.22	0.00	0.12	0.22	1.07
Animals	8.18	0.00	0.36	0.49	1.03
Accidents and physical damage	0.46	0.32	0.15	0.26	0.06
Sense of touch of objects	4.86	0.00	0.10	0.19	0.63
Chewing and biting objects	4.98	0.00	0.06	0.13	0.77
Moral	25.48	0.00	0.36	0.48	2.07
Fingernails	4.58	0.00	0.05	0.12	0.72
Fabrics	4.44	0.00	0.04	0.11	0.70
Food	8.02	0.00	0.21	0.33	1.09
Smells	11.91	0.00	0.28	0.40	1.43
Shivering	6.53	0.00	0.28	0.41	0.82
Goose bumps	5.25	0.00	0.19	0.30	0.67

*LLCI, lower level 95% confidence interval, ULCI, upper level 95% confidence interval*.

### Discussion

Grima is predominantly generated by high-pitched and squeaking noises. In fact, noises and squeaking, as well as scratching or touching with fingernails and scratching or touching of surfaces were exclusively mentioned as features of *grima*. Thus, the most frequent features of *grima* referred to either hearing (noise of chalk, 38%) or touching (scratching or touching with fingernails, 29%; scratching or touching of surfaces, 17%). In contrast, other stimuli such as accidents and physical damage, rotten food or those pertaining to the moral domain were both mentioned for *grima* and *disgust* (e.g., Haidt et al., 1994; Rozin et al., 1999).

With respect to the features mentioned as examples of physical reactions to *grima*, shivers and goose bumps constitute the most typical features of *grima*. In all, *grima* includes distinctive features related to auditory aversion, but also shares some features with other forms of aversion typically related to *disgust* (e.g., physical damage).

## Study 3: Physiological Correlates of *Grima*

In Study 2, participants reported different physical reactions as typical features of *grima* as compared to *disgust*. Whether or not this reported conceptual characterization is supported in terms of a parallel differentiation of physiological responses elicited by auditory elicitors of grima and disgust was the aim of Study 3. Moreover, we aimed at complementing the self-report data from the previous study with indices of physiological arousal during emotional episodes in order to obtain information about processes outside the participants’ self-reports (Lang, 1980). Therefore, we assessed the physiological activity changes in HR and SCR during the presentation of the most typical elicitors of grima: high-pitched, squalling sounds.

The engagement of defensive (and appetitive) physiological systems has been reliably related to a transient increase in sweat gland activity, thus often regarded as a measure of arousal (Lang and Davis, 2006). With respect to the temporal changes of HR in response to an external stimulus, the temporal changes often show an initial phase of HR deceleration, interpreted as orienting toward a novel and potentially threatening stimulus (Bradley, 2009). The subsequent HR acceleration is seen as indicating readiness and preparation for action in response to the event, and it increases with behavioral relevance of the stimulus (Moratti et al., 2006). In the context of disgust, these measures have been taken as indexing avoidance or rejection behavior (Vrana, 1993). Therefore, we aimed at analyzing whether disgust and grima show similar physiological profiles or if they vary in their access to basic defensive and arousal systems and therefore show different response profiles.

### Materials and Method

#### Participants

Thirty-seven German and American college students participated for class credits or a small financial bonus. The mean age was 24 years (*SD* = 2.9).

#### Stimuli and Design

A total of 29 stimuli were selected from the “International Affective Digitized Sounds” (IADS; Bradley and Lang, 1999), based on pleasure and arousal ratings. Eight stimuli were rated as unpleasant and disgusting (i.e., eliciting disgust, such as vomiting) and seven stimuli each were rated as pleasant (e.g., laughing), neutral (e.g., rain), and unpleasant (e.g., screaming), respectively, with pleasant and unpleasant not being different in emotional arousal. Because the IADS-system did not have specific grima-stimuli, we added 8 high pitched, squalling sounds that were indicated as eliciting grima by three independent Spanish raters in a pilot study. This resulted in a total of 37 stimuli. All stimuli were normalized to have unit energy (defined as the root mean square across their total duration of 6 s).

#### Procedure

Following the procedure reported by Bradley and Lang (1999), each trial began with the presentation of the message “Please prepare yourself for the evaluation of the next sound.” Next, one of the stimuli was presented together with a brief description and participants rated their experienced feelings on the 9-point valence and arousal scales in the paper and pencil version of the “Self-Assessment Manikins” (Bradley and Lang, 2000). Rating scores were averaged within each condition, for each participant, resulting in mean ratings of hedonic valence and emotional arousal for each of the 5 stimulus categories (i.e., grima, disgust, pleasant, neutral, unpleasant).

Heart rate was measured from electrocardiogram recorded with a BioPac bioamplifier using three disposable snap electrodes. Sensors were placed at the medial left and right forearms, and the electrocoardiogram was digitized at a rate of 200 Hz, constrained by filters between 0.1 and 50 Hz. HR changes over time were obtained by detecting R-waves using a Schmitt trigger and converting inter-beat intervals into beats per minute (bpm) values for 0.5 s bins, as proposed by Graham (1978). This method uses weighting of the temporal distance of heartbeats to a given time bin (here: 0.5 s) to yield a continuous function of HR estimates for subsequent time bins. The mean baseline (averaged across 2 s prior to sound onset) bpm value was subtracted from the HR time series to result in a waveform reflecting HR change from the pre-sound baseline over time.

The SCR was simultaneously recorded using electrodes placed adjacently on the hypothenar eminence of the left palmar surface using standard 8 mm silver–silver chloride electrodes filled with 0.05-m NaCl paste. The signal was recorded with a BioPac skin conductance coupler calibrated to detect activity in the range of 0–40 micro-Siemens (μS). The amplitude of the SCR was calculated in half-second bins and was scored as the peak change value during the 6 s sound presentations with respect to a 1 s pre-stimulus baseline. The minimum of the first 2 s after sound onset was used as a measure of initial HR deceleration and the maximum in the range between 3 and 6 s indexed the subsequent acceleration phase.

### Results

Skin conductance maxima, HR deceleration and acceleration, hedonic valence, and emotional arousal ratings were entered into separate ANOVAs with the factor of Stimulus Category (grima, disgust, pleasant, neutral, unpleasant). Significant *F*-values were followed up by examining differences between pleasant, neutral, and unpleasant IADS stimuli using linear and quadratic contrasts (trend tests), and planned contrasts (*t*-tests) between the stimuli of grima and of disgust.

Self-ratings showed a pattern consistent with normative data: pleasant (*M* = 5.31, *SD* = 1.43) and unpleasant (*M* = 5.18, *SD* = 1.36) stimuli were associated with greater emotional arousal ratings than neutral (*M* = 4.38, *SD* = 0.94) stimuli, resulting in an overall effect of stimulus category, *F*(4,144) = 3.04, *p* = 0.02, $\eta_{p}^{2}$ = 0.08, and a quadratic trend for pleasant, neutral, and unpleasant stimuli, *F*(1,36) = 12.81, *p* < 0.01, $\eta_{p}^{2}$ = 0.26. Grima (*M* = 5.16, *SD* = 1.47) and disgust (*M* = 5.14, *SD* = 1.30) stimuli did not differ from each other, *t* < 1, or from emotionally arousing stimuli from the IADS (pleasant and unpleasant; *ts* < 1). Grima (*M* = 5.66, *SD* = 1.54) and disgust (*M* = 5.31, *SD* = 1.30) stimuli were also perceived to be of comparable unpleasantness (*t* < 1.2), with a category effect in valence ratings, *F*(4,144) = 3.20, *p* = 0.02, $\eta_{p}^{2}$ = .08, reflecting the linear decrease of pleasure across the three groups of means representing pleasant (*M* = 6.26, *SD* = 1.06), neutral (*M* = 5.84, *SD* = 1.29), and unpleasant (*M* = 5.36, *SD* = 1.29) stimuli, *F*(1,36) = 13.34, *p* < 0.01, $\eta_{p}^{2}$ = 0.27. Again, grima and disgust stimuli did not differ from unpleasant stimuli, *ts* < 1.

**FIGURE 1:**
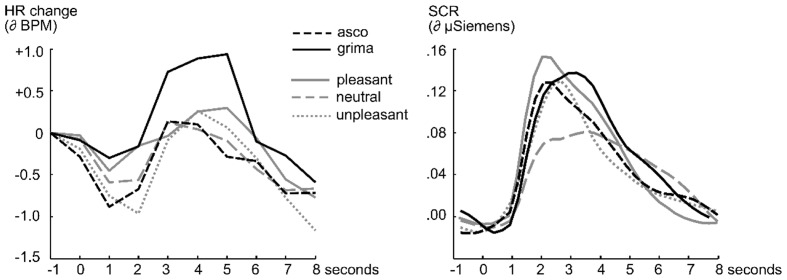
**Changes in heart rate (HR, **left**) and skin conductance response (SCR, **right**) during the presentation of disgust, grima, pleasant, unpleasant, and neutral sounds (Study 3)**. HR changes are represented in beats per minute (bpm) in 0.5 s bins intervals, while the SCR was calibrated to detect activity in the range of 0–40 micro-Siemens (μS). The time series cover the duration of the sound presentations and include a second prior to sound onset.

Heart rate data showed a more complex picture (see **Figure 1**, left panel), and a main effect of stimulus category, *F*(4,144) = 3.27, *p* = 0.01, $\eta_{p}^{2}$ = 0.08, indicating that greater initial deceleration of the heart was associated with greater unpleasantness across pleasant (*M* = –0.09, *SD* = 1.36), neutral (*M* = –0.48, *SD* = 1.30), and unpleasant (*M* = –1.02, *SD* = 1.20) stimuli, *F*(1,36) = 12.08, *p* < 0.01, $\eta_{p}^{2}$ = 0.25. Notably, grima (*M* = –0.16, *SD* = 1.56) stimuli did not follow this pattern and showed less deceleration than disgust (*M* = –0.80, *SD* = 1.14; 95% CI [–1.31, 0.02]), *t*(36) = 1.96, *p* = 0.06, *d* = 0.47, as well as unpleasant stimuli (95% CI [0.15, 1.57]), *t*(36) = 2.44, *p* = 0.02, *d* = 0.61, being at the same level as pleasant and neutral stimuli (*ts* < 1.1). Similarly, grima (*M* = 0.94, *SD* = 2.11) was different from disgust (*M* = 0.06, *SD* = 1.56; 95% CI [–1.73, –0.03]), *t*(36) = 2.10, *p* = 0.04, *d* = 0.47, in terms of the subsequent HR acceleration, where again an overall effect of stimulus category was observed, *F*(4,144) = 2.52, *p* = 0.04, $\eta_{p}^{2}$ = 0.07. Grima showed the greatest activation, exceeding neutral (*M* = –0.10, *SD* = 1.29; 95% CI [–1.90, –0.17]), *t*(36) = 2.43, *p* = 0.02, *d* = 0.59, but not pleasant (*M* = 0.52, *SD* = 1.70) and unpleasant (*M* = 0.28, *SD* = 1.61) stimuli (*ts* < 1.8). A quadratic contrast for the pleasant, neutral, and unpleasant stimuli replicated previous work with these sounds (Bradley and Lang, 2000), *F*(1,36) = 4.25, *p* = 0.05.

Skin conductance data evidenced a strong relationship with emotional intensity (arousal), leading to a category main effect, *F*(4,144) = 2.71, *p* = 0.03, $\eta_{p}^{2}$ = 0.07, and SCR to grima (*M* = 0.13, *SD* = 0.13) and disgust (*M* = 0.10, *SD* = 0.13, *t* < 1.1) stimuli were at the same level as those evoked by unpleasant (*M* = 0.12, *SD* = 0.15) and pleasant (*M* = 0.12, *SD* = 0.16) stimuli (all *ts* < 1.1). The only condition with significantly different SCRs was the neutral (*M* = 0.04, *SD* = 0.06) stimuli condition, resulting in a quadratic trend when comparing pleasant, neutral, and unpleasant categories, *F*(1,36) = 14.82, *p* < 0.01, $\eta_{p}^{2}$ = 0.92.

### Discussion

Stimuli aiming to convey grima or disgust fall into the range of unpleasant stimuli, evoking significant SCR increase and a typical pattern of HR deceleration/acceleration. Our procedure led to self-rating and reflex physiology results consistent with earlier work using auditory stimuli (Bradley and Lang, 2000). Importantly, grima and disgust showed significant differences in HR changes, but did not differ in affective ratings and skin conductance. This suggests that grima stimuli were as arousing as high arousing pleasant, unpleasant, and disgust stimuli. The specific defensive engagement (HR) characteristic of unpleasant and disgust stimuli (i.e., strong HR deceleration consistent with orienting) was not present with grima stimuli, which in contrast were associated with greater HR acceleration, consistent with less specific defensive orienting, but strong autonomous action readiness.

## Study 4: Self-Regulation of *Grima* Via Implementation Intentions

In a fourth study, we aimed at critically testing whether the differentiation between grima and disgust would also hold true when it comes to the regulation of one of them (e.g., downregulating grima, but not disgust). We devised a study based on implementation intentions (Gollwitzer, 1999), which are if-then plans that spell out when, where, and how a set goal has to be put into action by linking a critical situation with a goal-directed behavior: “If situation x is encountered, then I will perform behavior y!” Whereas mere goal intentions designate desired end-states the individual feels committed to attain (“I intend to reach z!”), implementation intentions refer to the realization of the goal intention and create a commitment to respond to a specified critical situation in a planned, goal-directed manner. Thus, implementation intentions are formed in the service of goal intentions.

Implementation intentions create a mental link between the specified critical situation (“if-part”) and the intended goal-oriented response (“then-part”). As a consequence, the mental representation of the critical situation becomes activated and is highly accessible (e.g., Parks-Stamm et al., 2007; Webb and Sheeran, 2007). Forming such “if-then” links also establishes a strong cue-behavior link (Webb and Sheeran, 2008), such that the presence of the specified cue automatically elicits the linked response (e.g., Bayer et al., 2009).

Implementation intentions have been consistently found to help people achieve their goals in several domains, be it promoting desired behaviors such as in the educational domain (e.g., attaining learning goals; Stalbovs et al., 2015), in the interpersonal domain (e.g., interest in sustained contact; Stern and West, 2014), or health-related goals (e.g., reducing pregnancy risk; Martin et al., 2011), and also help control unwanted habitual responses (e.g., reducing speeding; Brewster et al., 2015). They have also been shown to be effective for different kinds of populations, such as low-income and racial/ethnic minority populations (Greiner et al., 2014), as well as children with ADHD (Gawrilow et al., 2013), and adolescents or older people (Hall et al., 2014).

Though research on emotion regulation by implementation intentions was still rather scarce in 2006, when the first meta-analysis on the effects of implementation intentions was published (Gollwitzer and Sheeran, 2006), its relevance has grown considerably in the last years. Indeed, research has specifically shown that implementations intentions are effective in down-regulating emotional experiences such as disgust (e.g., Schweiger Gallo et al., 2009; Miles et al., unpublished manuscript, Study 1), fear (Schweiger Gallo and Gollwitzer, 2007; Schweiger Gallo et al., 2009, Studies 2 and 3), or anxiety (Varley et al., 2011).

Building on these findings, we hypothesized that if grima and disgust are two distinct emotional experiences, the emotion regulation elicited by forming implementation intentions should allow participants to down-regulate their grima-experience independently of the emotional reactions to disgust-eliciting sounds. Thus, if our assumption holds true, the specific down-regulation of grima by implementation intentions would confirm the existence of two distinct emotional experiences, as suggested by our previous studies.

### Materials and Method

#### Participants and Design

Forty-four Spanish college students (mean age = 22.56; *SD* = 3.87) listened to a total of 14 pleasant and unpleasant sounds from the IADS (Bradley and Lang, 1999), which were selected based on pleasure and arousal ratings from the Spanish adaptation by Fernández-Abascal et al. (2008). As the IADS-system did not have specific emotion-evoking sounds, we added ten sounds that had been judged as eliciting grima and disgust by three independent raters. The valence and arousal ratings were assessed by two different self-report Manikin scales (Bradley and Lang, 1994).

#### Procedure

Participants were randomly assigned to one of two conditions and were told that they would be asked to rate their emotional responses to each of the presented sounds. Participants in the mere goal intention condition (control condition) were asked to form the intention “I will not feel grima with the presented sounds!” Implementation intention participants also formed this goal and added the if-then plan: “And if I hear a grima-eliciting sound, then I will ignore it!” Next, participants were handed the paper and pencil version of the “Self-Assessment Manikins” (Bradley and Lang, 2000), and were familiarized with a typical trial, which began with the presentation of the message “Please prepare yourself for the evaluation of the next sound.” One of the sounds was then presented together with a short description of how it originated, and participants were advised to indicate their experienced feelings at the moment of hearing the sounds in less than 5 s. All participants were then given a final questionnaire that assessed how committed they felt to meeting the goal of down-regulating grima, as well as participants’ perceived performance. Both items were accompanied by 9-point answer scales ranging from 1 (“not at all”) to 9 (“very”).

### Results

#### Main Analyses

A 2 (self-regulation condition: goal intention condition, implementation intention condition) X 4 (type of sounds: pleasant, unpleasant, grima, disgust) ANOVA revealed no significant interaction effect of type of sound and self-regulation condition on valence, *F*(3,126) = 0.69, *ns*, nor on arousal ratings, *F*(3,126) = 1.02, *ns*. Further analyses also revealed that a one-factorial ANOVA on valence ratings (down-regulation: goal intention vs. implementation intention) yielded no significant self-regulation condition effects for the pleasant sounds, *F* < 1, the unpleasant sounds, *F*(1,42) = 2.66, *ns*, or for the disgust -eliciting sounds, *F*(1,42) = 1.54, *ns.* Importantly, there was a marginally significant effect for the grima-sounds, *F*(1,42) = 3.96, *p* = 0.053, *d* = 0.61, with implementation intention participants rating the grima-sounds as less unpleasant than goal intention participants (*M* = 6.54, *SD* = 1.11 and *M* = 7.22, *SD* = 1.13). In line with our hypothesis, the one-way ANOVA for the grima-eliciting sounds was also significant for the arousal ratings, *F*(1,42) = 5.46, *p* = 0.02, *d* = 0.72. Specifically, grima-sounds were rated as less arousing by implementation intention participants than by goal intention participants (*M* = 4.99, *SD* = 1.93 and *M* = 3.77, *SD* = 1.42). Likewise, no differences between conditions were found for pleasant sounds and disgust-eliciting sounds (*Fs* > 0.1), nor for unpleasant sounds, *F*(1,42) = 1.21, *ns*.

Hence, only participants who furnished their goal intention to not feel grima with a respective implementation intention rated the sounds as less unpleasant and less arousing than goal intention participants. Importantly, this effect of implementation intentions on valence and arousal ratings only held true for the grima-eliciting sounds, but was not observed for disgusting sounds (i.e., implementation intention participants were able to differentially down-regulate the targeted emotion).

#### Post-experimental Questionnaire

Goal intention and implementation intention participants did not differ with respect to how committed they felt to their goal intention (*M* = 7.20, *SD* = 1.77 and *M* = 6.67, *SD* = 2.04; 95% CI [–0.01, 1.36]), how much they tried to achieve this goal intention (*M* = 7.40, *SD* = 1.85 and *M* = 7.58, *SD* = 1.79; 95% CI [–1.29, 0.93]), how well they succeeded in realizing their goal intention (*M* = 6.70, *SD* = 1.53 and *M* = 7.04, *SD* = 2.14; 95% CI [–0.01, 1.36]), or how much they visualized the instructions (*M* = 6.55, *SD* = 2.04 and *M* = 5.83, *SD* = 2.71; 95% CI [–1.49, 0.81]), all *ts* < 1. Thus, the observed effects are not based on a higher commitment or a higher perceived performance.

### Discussion

Further preliminary evidence for the differentiation between the components of *grima* and *disgust* is provided in this study, where participants formed either a goal intention or an implementation intention to down-regulate their emotional reactions to grima-eliciting sounds. Results showed that implementation intentions that targeted grima only managed to affect participants’ grima-related responses, but not the responses evoked by the other pleasant and unpleasant sounds, including those evoking disgust. As participants were able to selectively down-regulate grima, as compared to disgust, this study also suggests that the experience described by ‘grima’ is not a reflex in the sense of a fast and inevitable response to specific stimuli (Konorski, 1948) but rather points to a differentiation of grima and disgust as two distinct emotional experiences.

One might be tempted to assume that implementation intention participants reported having down-regulated their emotional reactions because they received instructions to do so. If this were true, both goal intention participants and implementation intention participants should have reported lower grima-feelings. However, participants in the goal intention condition reported feeling grima, whereas participants in the implementation intention condition did not. Still, one might want to argue that implementation intention participants experienced stronger experimenter demand as they are instructed to down-regulate their grima-feelings by using implementation intentions. In this regard, Schweiger Gallo et al. (2009) found no differences in the experimenter demand experienced by goal intention versus implementation intention participants in their studies on the self-regulation of disgust and fear. This suggests that the differences they observed between the goal intention condition and the implementation intention condition in down-regulating disgust and fear were due to the higher self-regulatory effectiveness of implementation intentions as compared to goal intentions, rather than on differential experimenter demand.

## Study 5: Cross-Cultural Comparison of the Categorization of *Grima*

In Study 5, we asked whether the negative affective experience caused by aversive sounds is sufficiently distinctive to be referred to by using a unique term in other Western countries than in Spain. As people seem refer to it in English and in German using different expressions, we asked whether this would also hold true when asked to characterize the elicitors of different emotions. Thus, we utilized a procedure developed by Boucher and Brandt (1981), aimed at testing cross-cultural similarities and differences in the categorization of affective reactions. We expected coherent descriptions of *happiness*, *anger*, *fear*, *sadness*, *shame*, *guilt*, and *disgust* in English, German and Spanish, as well as of *grima* in Spain, but no specific cross-cultural comparison of *grima* in Germany and the USA.

### Pilot Study

Forty-seven students from a US University, 54 Spanish participants and 10 students from a German University were asked to describe a situation which typically evokes *anger* (*ira*, *Ärger*), *disgust* (*asco*, *Ekel*), *fear* (*miedo*, *Angst)*, *happiness* (*alegria*, *Freude*), *sadness* (*tristeza*, *Traurigkeit*), *shame* (*vergüenza*, *Scham*), *guilt* (*culpa*, *Schuld*), and *envy* (*envidia*, *Neid*). Two independent raters chose the three most characteristic descriptions for each of them among all the answers.

### Materials and Method

#### Participants and Design

One-hundred and sixteen German (mean age = 22.65; *SD* = 5.62), 85 Spanish (mean age = 21.91; *SD* = 3.36), and 99 US participants (mean age = 19.46; *SD* = 1.45) indicated which emotion was evoked by the situations selected among the descriptions obtained in the pilot study. We also included three typical elicitors of the auditory aversive reaction of *grima* previously identified in Study 1 (scraping fingernails on a blackboard, the noise of a chalk on a blackboard, and an open bone fracture). We relied on an open-ended response format and instructed participants to write down which emotion was evoked primarily (e.g., *happiness* or *sadness*) rather than how the emotion was (e.g., unpleasant).

### Results

American and German participants resorted to different linguistic labels to refer to the aversive reaction evoked by scraping fingernails on a blackboard and the noise of a chalk on a blackboard (see **Table 2**). In fact, they labeled this experience as ‘disgust’ and ‘Ekel’. In contrast, the elicitors of other categories of emotions were accessible and accurately identified, including *disgust* (e.g., unclean, clogged toilet was identified by 93% of German participants; seeing someone vomit was identified by 69 % of American participants), *happiness* (e.g., spending time with friends was identified by 84% of German participants and 78% of American participants), and *sadness* (e.g., death of a beloved person was identified by 86% of German participants; having to put their dog down was identified by 73% of American participants). In the same vein, Spanish speaking individuals’ characterization of the elicitors of *happiness*, *shame*, or *fear* were also accurate (e.g., seeing somebody you like and haven’t seen for a while, screwing things up in front of a large group of people, and feeling that somebody is following you on the street were identified by 87, 86, and 74% of Spanish participants, respectively). Importantly, however, Spanish speaking individuals also distinguished between *grima* and *disgust* (e.g., finding vomit on the floor was identified as *disgust* by 89% of Spanish participants.)

**Table 2:** Percentage of features mentioned by at least 10% of the participants in Germany, the USA, and Spain for grima elicitors (Study 5).

Antecedent	Term in Germany	Term in the USA	Term in Spain
Scraping fingernails on a blackboard	‘Ekel’ (41%), ‘Grausen’ (11%)	‘Annoyance’ (15%), ‘Anger’ (15%), ‘Irritation’ (15%)	‘Grima’ (66%)
Chalk on a blackboard	‘Ekel’ (40%), ‘Schauder’ (11%)	‘Annoyance’ (22%), ’Irritating’ (14%), ‘Discomfort’ (11%)	‘Grima’ (60%)
Seeing an open bone fracture.	‘Ekel’ (58%)	‘Disgust’ (46%)	‘Grima’ (38 %), ‘Asco’ (26 %)

*‘Ekel’ is the common translation for ‘disgust’, whereas ‘Grausen’ can be translated as ‘horror’ and ‘Schauder’ as shudder or shiver*.

### Discussion

Study 5 points to an aversive experience which is reliably labeled as ‘grima’ in Spanish. Importantly, in those languages in which there is no specific term to characterize the aversive reaction to stimuli associated to *grima*, this experience is described being related to the term of ‘disgust’ (or ‘Ekel’ in our German sample) along with other aversive reactions. Thus, both English and German speakers bridge what seems to be a lexical gap by using semantic neighbors. In contrast, ‘grima’ and ‘asco’ were used to refer to different aversive reactions by Spanish speaking individuals, with the exception of “seeing an open bone fracture”, which was identified as *disgust* by English and German speakers, but was characterized by Spanish speakers as eliciting *grima*, and, to a lesser extent, *disgust*.

## General Discussion

The present research has attempted to characterize the everyday concept behind an aversive response, which is known in Spanish as ‘grima’. Contributing to the literature on folk concepts and their differences across languages, our studies point out that the everyday concept of *grima* differs from *disgust* (Study 1), both with respect to its typical elicitors and physiological concomitants (Study 2). In line with these results, the findings of Study 3 show that the typical elicitors of *grima* have different effects on HR. Study 4 provides further evidence for the conceptual and experiential differentiation between *grima* and *disgust*. Extending previous research, where the regulation of emotional reactions by implementation intentions was based on visual stimuli (for example, Schweiger Gallo et al., 2009), we used auditory stimuli to show that participants who formed a grima-related implementation intention were able to selectively down-regulate their emotional experience to the grima-eliciting sounds.

Interestingly, German and US English speakers do not have a comparable lexical analog for ‘grima’ (Study 5). In fact, *grima* goes nameless in these languages. This is even more remarkable, as closely related Indo-European languages such as English, German and Spanish differ with regards to the linguistic label used to refer to this aversive reaction. In fact, 11% of our German sample used the term ‘Schauder’, which can be translated as ‘shudder’ or ‘shiver’, to refer to this experience. Though they do not have a distinct term to refer to the experience, they seem to recognize it and use the physiological concomitants to refer to its corresponding affective reaction.

### Implications

Our research is inspired by Russell (1991, p. 428), who stated: “I take it for granted that psychologists are interested in the emotions of all people, not just those who speak English”. By focusing on an emotion concept which is present in a Western culture, our studies complement and support the ethnographic evidence reviewed by Russell mostly for non-Indo-European languages. The importance of *grima* in the Spanish everyday knowledge of emotion suggests that the English emotion lexicons might not only fail to adequately represent the emotional life of non-Western individuals (see Scollon et al., 2004), but also of some Western individuals.

Is the aversive experience labeled as ‘grima’ a mere reflex reaction? Reflexes are traditionally defined as simple, direct, quick, and inevitable responses to specific eliciting stimuli (Konorski, 1948). Many reflexes may also be characterized by fixed, brief latencies and thus are considered not amenable to voluntary suppression, although they can be modulated by factors such as learning/experience and motivational state (Lang et al., 1990). In line with research on emotion regulation by implementation intentions (Webb et al., 2012), the findings of Study 4 suggest that grima can be down-regulated by forming implementation intentions and thus might not have to be considered a reflex. Indirect support for the assumption that the Spanish term ‘grima’ refers to an emotional experience rather than a reflex can also be found in our second study, were grima was reported to be elicited by different kinds of stimuli (not just scratching or touching of surfaces). Grima is also more than a set of bodily reactions as, for example, “the chills” are. “Chills” have been characterized, for example, by Maruskin et al. (2012), who found that this sensation is composed by goosetingles and coldshivers. In contrast, grima includes a variety of elicitors and the experience of grima seems to also involve cognitive processes, as participants in the fourth study who formed an implementation intention were able to down-regulate the grima experience, but not the disgust experience. Even more so, the appraisal of grima differed from the appraisal of disgust. Thus, if we accept that emotions require cognitive appraisals, grima cannot be regarded as a mere reflex or a set of bodily responses. These observations are also in line with data obtained when we asked Spanish participants whether grima and disgust are emotions: participants considered that grima was an emotion to an even greater extent than disgust. Future research might want to address these issues in more detail and further dig into the characterization of *grima*.

Is grima a distinct emotion? There are various possible answers to this question, depending on which of the different approaches one adheres to. A first potential interpretation is that the concept of *grima* is a natural kind which reflects the structure of a universal basic emotion with its specific experiential, physiological and behavioral constituents. In support of this view, the results of Study 3 showed that participants who displayed a differentiated physiological reaction to grima elicitors were German students who, as shown in Study 5, lack a category of emotion similar to *grima* and assimilate *grima* into other categories of emotion such as *disgust*. From this perspective, *grima* is just a member of one of the “families” of basic emotions recently described for example by Ekman and Cordaro (2011) or Levenson (2011). While this approach is intuitively simple, it raises a number of objections. The most important problem, from a theoretical point of view, is that it lacks parsimony. The absence of a definitive list of basic emotions (Ortony and Turner, 1990) allows the basic-emotion theory to include all kinds of variations and post-hoc sub-categories of basic emotions, in an evolution that has manifest epistemic similarities with the drift of the instinct theories at the beginning of the twentieth century. Any undescribed emotional experience should be assimilated to a basic emotion family, regardless of its differential characteristics.

A second, in our view more appropriate approach to our findings is inspired by psychological construction models (for example, Russell, 2003; Barrett, 2013), but is also compatible with appraisal theory. Based on this approach, *grima* is a native everyday concept of emotion alluding to a set of potentially universal affective (e.g., core affect), cognitive (e.g., appraisals), and behavioral processes (e.g., action tendencies). This set is what, in terms of psychological constructionism, is called an “emotional episode.” In some cases, these coincidental processes are categorized, in terms of everyday language, as an emotion with its corresponding term in that language. The existence of the term probably enhances the extent of awareness about the emotional experience in a sort of “soft version” of the Whorf hypothesis, but the existence of an everyday concept is not a necessary prerequisite for experiencing the emotional episode. If there is no everyday concept, the emotional episode is not labeled by the subject, or it is assimilated to the everyday concept of a roughly similar experience (e.g., *disgust*, in the English-speaking participants).

### Limitations and Future Directions

An important concern, already addressed in other studies on the everyday knowledge of emotions (see Russell, 1992; Hurtado de Mendoza et al., 2010), is that our approach might miss the objective definition of *grima* because it focuses on the potentially accidental definition provided by the participants (i.e., their everyday concept). We admit that the current approach does not allow to determine all of the necessary and/or sufficient affective, cognitive, and behavioral components that cause the experience of grima. However, the isolation of the affective, cognitive, and behavioral components requires, first of all, the description of the everyday concept of *grima*. In this regard, the best descriptions of everyday concepts of emotion are prototypical, probabilistic representations The most typical features of the concept are not necessarily the most important objective affective, cognitive, or behavioral components of grima, but those that constitute the subjective cognitive representation of grima, allowing for distinguishing between those individuals who consistently report grima and those who do not do so; a prerequisite for a further objective description of the emotional episode behind the everyday concept of *grima*.

Another important, and related limitation of our studies is that they did not include a systematic causal test of the elicitors of grima. In this respect, the most intriguing question concerns the origin and function of this specific reaction to certain sounds. A set of affective, physiological and behavioral aversive reactions to some specific auditory stimuli is probably shared by all humans. In fact, the existence of a form of auditory aversion to specific sounds has received some attention from neuroscience (e.g., Schönwiesner and Zatorre, 2008), as well as from research with animals, for example with non-human primates (McDermott and Hauser, 2004). The reasons underlying the aversive experience evoked by scraping sounds remain nevertheless to be researched (Cox, 2008). One possibility relates to the potential adaptive benefits of this experience, as these sounds might be associated with dangerous predators or objects, and could thus serve to avoid the dangers derived from either of them. However, the results of Study 3, where a heightened action readiness but not defensive orientation was found, suggest a need to get rid of the unpleasant experience, but not to flee from the stimulus. These follow-up questions need to be analyzed within a systematic program of research.

Why *grima* became a more salient emotional episode in Spanish than in other languages is an unresolved question in our studies. The etymological origins of ‘grima’ are not clear; a potential origin in Old German is the term ‘grimm’ (‘horrible’) but this root does not explain the current meaning of the term. Importantly, the linguistic label that identifies this affective reaction (i.e., ‘grima’) as a unitary phenomenon is only found in some languages or, at least, in Spanish. As has been observed for other populations and emotion terms such as Tahitian society, that lacks a term for *sadness* (Levi, 1984 in Russell, 1991), or the Rarámuri Indians, who lack a word for *guilt* (Breugelmans and Poortinga, 2006), there is dissociation between a shared underlying affect system and its cultural representation. The role of culture and language on human perception has received attention in recent years. For instance, language has been found to play an important role in color recognition and evaluative judgments (Roberson et al., 2000), as well as smell (Wnuk and Majid, 2014). Indeed, recent research on cross-modal associations of odor and color found that language plays an important role in odor-color associations (De Valk et al., 2016). Further, it has also been questioned whether the categorization of human emotional facial expressions depends on the existence (or not) of linguistic labels. Whereas it has been claimed by some researchers that facial expressions are perceived categorically even if there are no lexical categories which differentiate between *anger* and *disgust* (Sauter et al., 2011), others have questioned the assumption that categorical perception is entirely universal (e.g., Roberson et al., 2010). Therefore, an intriguing sequel to the present research could focus on the potential differences in the cognitive representation, as well as the affective processing of the aversive experience of grima through a comparison of speakers, with and without a specific linguistic label for this experience. Another important question deserving attention is whether the emotional episode referred by the term ‘grima’ requires not only some specific behavioral, experiential or physiological components but also the mental representation of the emotional episode through a specific conceptual category. However, addressing this question is beyond the scope of this article.

## Ethics Statement

Study 1: Participants tacitly gave their consent by completing and returning the questionnaire.

Study 2: Participants tacitly gave their consent by completing and returning the questionnaire.

Study 3: Participants gave written informed consent after being informed of the nature of the study. The study was approved by the internal review boards of the University of Konstanz, Germany, and the University of Florida, USA.

Study 4: Participants gave written informed consent after being informed of the nature of the study.

Study 5: In the pilot study, participants tacitly gave their consent by completing and returning the questionnaire. Participants gave their consent after being informed of the nature of the study. The study was approved by the internal review boards of New York University, USA, and the University of Konstanz, Germany.

Three of the five studies are based on questionnaires. These questionnaires were either distributed by a snowball system to acquaintances or in the classroom. In the latter case, as the questionnaires were completed anonymously, those who chose or did not choose to participate could not be identified. Thus, participants tacitly gave their consent by completing and returning their questionnaires. Approval by the Internal Review Boards was obtained in line with the customs of the participating institutions.

The involvement of minors was not targeted in our study. Indeed, only a negligible number of 17 years old filled out a questionnaire.

## Author Contributions

IS and JF formulated the research question. IS, JF, PG, and AK designed the studies. IS and AK collected data. IS, JF, and AK analyzed the experimental results. IS wrote the article and all authors were involved in editing and revising the early drafts and approving the final manuscript.

## Conflict of Interest Statement

The authors declare that the research was conducted in the absence of any commercial or financial relationships that could be construed as a potential conflict of interest.

## Footnotes

1 In order to clarify when we are speaking about a term of emotion versus the conceptual category of the emotion we use italic characters when alluding to a conceptual category or its features (e.g., *vergüenza*, *shame*), and apostrophes when alluding to the term (e.g., ‘shame’). When we talk about an emotion in a general, unspecific way we do not use special characters (e.g., shame).

2 In some of the studies using auditory stimuli, the aversive experience, which is a central element of the concept of *disgust*, is not produced directly, but through the evocation of a visual stimulus typically categorized as *disgust*, which in turn produces the aversive reaction. This meta-experience is for example induced by the sound of a vomiting person (e.g., Croy et al., 2013; Seidel and Prinz, 2013). Some theorists have suggested that such auditory stimuli may not evoke disgust directly, but rather engage associative networks including other features of vomit, such as its appearance and smell (Lang, 1979; Chapman and Anderson, 2012).
